# A multi-strain human skin microbiome model provides a testbed for disease modeling

**DOI:** 10.3389/frmbi.2025.1473292

**Published:** 2025-02-04

**Authors:** Angela L. Maloney, Tyler Crawford, Jordan Hurlbut, Monica Martinez, Thomas J. Mulhern, Elizabeth L. Wiellette, Else M. Vedula, Vidhya Vijayakumar

**Affiliations:** Bioengineering Division, Draper, Cambridge, MA, United States

**Keywords:** skin, microbiome, consortium, atopic dermatitis, organ-on-chip, *in vitro* model

## Abstract

The skin microbiome plays a critical role at the interface between the human epidermis and the environment, providing colonization resistance against pathogenic strains, training host immunity, and supporting epithelial turnover. Inversely, dysbiotic skin microbiome states are associated with skin disease, particularly inflammatory conditions such as atopic dermatitis and psoriasis. Current evaluation of human host and microbiome interactions relies on *post hoc* studies after disease onset. This limits the ability to evaluate the causal roles of host and microbe during disease progression. One approach to characterizing microbial and host biology in a controlled and reproducible context is to derive *in vitro* models of sufficient complexity and stability to support perturbation and response. Current tools for studying these processes are focused on testing antagonistic or synergistic relations between two or more strains for short (hours to days) culture durations, thereby precluding studies of relevant complexity and chronic disease states. Here, we present an *in vitro* model of the human skin microbiome comprising a six strain consortium colonizing primary human keratinocyte-derived tissue in Air-Liquid Interface for up to 7 days. We evaluated readouts of tissue health including histology, gene expression, and transepithelial electrical resistance (TEER), as well as relative strain abundance to characterize microbiome stability over time. Skin cells formed a complex tissue structure over two weeks and maintained stable or increasing TEER after 7 days of co-culture with the microbial consortium. Up to five of the six strains were viable on the skin tissue surface on day 7 as validated by custom qPCR assays, demonstrating a robust and stable testbed for microbiome studies. A remarkable feature of this model is the persistence of *Cutibacterium acnes* in an aerobic tissue culture environment, since *C. acnes* growth is typically demonstrated under anaerobic conditions, suggesting that the skin tissue model is conducive to more natural growth states of native skin strains. The addition of cytokines representative of atopic dermatitis elicited a marked decrease in tissue barrier by day 7 compared to healthy controls, irrespective of the microbiome presence. Furthermore, an alteration in relative strain abundance was observed in diseased model tissues, demonstrating capability to study the impact of disease states on the microbiome and vice versa. We envision this model system as a test bed to evaluate the influence of commensals on host biology, the influence of external environment on microbiome stability, and chronic diseases impacted by dysbiosis.

## 1 Introduction

Human skin health relies in part on a partnership between the host epidermis and the resident microbiota, a diverse community of microbes composed of bacteria, fungi, and viruses. Together, the host and microbes maintain a homeostatic tissue barrier that protects the body from the external environment (Flowers and Grice, 2020; Swaney and Kalan, 2021; Harris-Tryon and Grice, 2022). The outer layer of the skin, the epidermis, provides a supportive environment for commensal microbes, which in turn delivers a defense against pathogen colonization, trains the immune system, supports wound healing, and promotes dermatological health (Matsui and Amagai, 2015; Naik et al., 2015; Rosso et al., 2016; Belkaid and Harrison, 2017; Linehan et al., 2018; Uberoi et al., 2024). Despite the harsh, acidic environment of the skin surface, colonizing microbes thrive in a commensal community, which includes ubiquitous bacteria like S*taphylococcus epidermidis* and highly prevalent *Cutibacterium acnes*, an anaerobic bacteria of the skin microbiome (Conwill et al., 2022; Severn and Horswill, 2023) (Conwill et al., 2022). Recent advances in sequencing technology and longitudinal sampling studies have provided a general recognition that skin health and resilience are predominantly indicated by microbial complexity and longitudinal stability (Oh et al., 2016; Byrd et al., 2018).

While they typically support healthy skin biology, the microbiota can become dysbiotic, or imbalanced, either by introduction of a pathogenic strain or by a change in metabolism or composition of the microbiome. It has been well documented that a dysbiotic skin microbiome is often correlated with inflammatory skin diseases such as atopic dermatitis (AD), psoriasis, and acne, further emphasizing the synergistic relationship between mammalian and microbial cells of the human skin (Lee and Kim, 2022; Severn and Horswill, 2023). For instance, in the case of AD, the relative abundances of *Staphylococcus aureus* and *S. epidermidis* are elevated compared to healthy or non-flared skin (Byrd, 2017; Kong et al., 2012; Fyhrquist et al., 2019). Due to the interdependency between host inflammatory status and microbial metabolism, it is challenging to disentangle causal and response effects after disease onset (Harris-Tryon and Grice, 2022). Typically, evaluation of the causative role of a particular bacterial strain is carried out through correlation of a strain or metabolite with disease state, isolation of the candidate strain, and evaluation on an animal model for disease induction (Li et al., 2006; McIntyre et al., 2016). However, animals are particularly distinct from humans in immune profile and microbiome composition and do not always provide a relevant background microbiome or host immune response (Ross et al., 2018; Bjornson-Hooper et al., 2022). Alternatively, microbial cells or extracts can be evaluated directly on human cells *in vitro* for reactivity. This strategy can be successful to characterize host cell activation, but current microbiome models lack the complexity to test the converse causality of host factors on a balanced microbiome (Kang et al., 2015; Nakatsuji et al., 2017)

There is a need to develop and improve representative, *in vitro* models of human skin tissue and its resident, complex microbiota (Smythe and Wilkinson, 2023). Such model systems should readily enable investigation of host-microbiome interactions in healthy states, the induction of pathogenicity, and the causes and treatments of skin diseases. Complex human skin reconstructions are available, including commercial versions (Labskin, EpiDerm) as well as cultured biopsies collected directly from human skin, which are typically sterilized (De Wever, 2015; Hardwick et al., 2020; Hofmann et al., 2023). A model that incorporates a commensal consortium of microbes in a stable co-culture with host tissue, with demonstrated feedback signaling between host and the resident microbes would fill existing gaps in microbiome toolkit to study host-microbiome interactions. Ideal representations of the human skin and its microbiota require both microbial diversity and stable longevity, features which will together provide a reliable and predictive context for evaluating host-microbe symbiosis and its disruption.

As a step towards a complex *in vitro* host-microbiome model, we present here a human skin microbiome testbed, SURFACE (Skin microbiome (µbiome) Reconstruction For Assessment of Cutaneous Effects), which supports a multi-strain bacterial culture on a human skin equivalent model. Combinatorial screening of candidate human skin commensal bacterial strains identified six strains and inoculation concentrations; resulting consortia were applied to mature skin tissues cultured at air-liquid interface (ALI). Direct quantification of the microbes periodically during the co-culture demonstrated that the strains could colonize and persist for 7 days, including the anaerobic strain, *Cutibacterium acnes*, and the pathobiont species *Staphylococcus epidermidis*. We also demonstrate that the SURFACE platform can be used as an appropriate model of host-microbiome response to a disease state. When exposed to Atopic Dermatitis (AD)-associated cytokines, the host tissue loses barrier function and secretes inflammation-relevant cytokines. Strikingly, the host response is paired with consistent modulation of the six-strain microbial consortium, which loses diversity similar to changes in AD patient microbiomes (Kong et al., 2012; Kim and Kim, 2019). These results present an important advance in models of host-microbiome interactions and can support the future evaluation of microbiome responses to pathogens, toxins, environmental changes, and introduction of engineered microbes.

## 2 Materials and methods

### 2.1 Skin tissue culture

Primary human keratinocytes (Normal Human Epidermal Keratinocytes; NHEK) were purchased from Lifeline Cell Technology (Frederick, MD). NHEKs were expanded in CnT-PR (CellNTec) and cryopreserved for seeding. On the day of seeding, noted as Day -7, PET Transwell inserts with 0.4 micron pores and a 0.33 cm^2^ growth area (Corning) were coated in 50 µg/mL (5 µg/insert) of human plasma fibronectin (Millipore) for 1 hour at 37°C. Cryopreserved NHEKs were thawed quickly, added to 10x volume of fresh media, and centrifuged at 150 X g for 4 minutes to remove cryopreservation agents. Media was aspirated from the cell pellet and resuspended to 250,000 cells/mL in CnT-PR-FTAL5 (CellNTec). Cells were seeded into inserts at 50,000 cells/insert and maintained in submerged culture for 3 days. Media was changed every other day.

On the fourth day of submerged culture, Day -3, TEER was measured using the EVOM-3 (World Precision Instruments) and STX-III electrodes (World Precision Instruments). Media was aspirated from apical and basal chambers and replaced with only 300 µL of media in the basal chamber to begin ALI culture. NHEKs were allowed to differentiate at Air Liquid Interface (ALI) conditions for 3 days before bacterial inoculum was introduced on Day 0, with basal media being refreshed every other day. For AD disease model, basal media was supplemented with 10ng/ml of rhIL-22, rhTNF-α, rhIL-4 and rhIL-3 (R&D Systems) on Day 0 of bacterial inoculation and refreshed every other day until takedown.

At takedown, on either Day 4 or Day 7, media was added to apical and basal chambers to collect TEER. TEER measurements were normalized by subtracting the blank TEER value of Transwell and multiplied by the surface area of the Transwell insert. TEER is represented as mean and standard deviation of multiple Transwells (n≥3 per condition) within a timepoint for a single experiment.

### 2.2 Bacterial strains and inoculation

Bacterial strains were purchased from ATCC and grown in indicated agar conditions atmosphere prior to inoculation (**Table 1**). All strains were lifted from agar plates and resuspended in TSB (BD Biosciences). The Optical Density (OD_600_) was measured using a NanoDrop (Thermo Scientific NanoDrop 2000c Spectrophotometer) and diluted until an OD_600_ of 0.1 was achieved. The bacterial solution was pelleted and then resuspended in twice the original volume of FTAL media for the six inoculated strains: *S. epidermidis*, *C. acnes*, *S. thermophilus*, *S. hominis*, *R. dentocariosa*, and *C. striatum*. All strains except *S. epidermidis* were diluted further to 1:500 and *S. epidermidis* was diluted 1:50,000. Single strain suspensions were plated, and Colony Forming Units (CFU) counted to determine the initial composition of the consortia that had been applied to the tissue.

**Table 1:** Strains used in this study, along with culture conditions and identification numbers.

Strain	Agar	Atmospheric Conditions	ATCC ID
*S. epidermidis* FDA strain PCI 1200	Tryptic Soy Agar	Aerobic	12228
*C. acnes* 417/52 [VPI 0391]	Tryptic Soy Agar +5% Sheep Blood	Anaerobic	11828
*S. thermophilus* (LMD-9)	S. thermophilus Agar Hi Media	5% CO2	BAA-491
*S. hominis* (NCTC 11320)	Tryptic Soy Agar	Aerobic	27844
*R. dentocariosa* CDC X599 [XDIA]	Tryptic Soy Agar +5% Sheep Blood	Aerobic	17931
*C. striatum* NCTC 764 [IFO 15291]	Tryptic Soy Agar +5% Sheep Blood + 0.1% Tween80	5% CO2	6940

To prepare the final mixed inoculum, each individual bacterial dilution was combined in equal volume. 100 µL of the mixed strain inoculum was then applied to the apical side of the SURFACE model. The tissue was incubated for two hours at 37°C, 5% CO_2_ to allow the bacteria to engraft to the tissue. The excess inoculum was removed, and tissues were washed twice with 200 µL of Full Thickness media, restored to ALI, and returned to 37°C, 5% CO_2_. Microbiome replete skin tissue was maintained in ALI for up to 7 days, with the media in the basal chamber being changed every other day. Transwell replicates were taken down for evaluation at either day 4 or day 7 and compared to uninoculated Transwell tissue replicates. Each figure represent data from a single experiment.

### 2.3 Quantitative PCR for strain specific assay validation

To produce single strain gDNA template for qPCR standard curve generation, a modified protocol of New England Biolab’s Genomic DNA Purification Kit (NEB #T3010) was utilized on each strain used in the study. The standard protocol for Gram-positive bacteria was used with two modifications – During lysozyme treatment, additional enzymes were added for improved cell lysis (10 µL lysostaphin at 10 mg/mL and 10 µL mutanolysin at 5 mg/mL). Two freeze thaw cycles in liquid nitrogen were incorporated prior to spin column extractions. The extracted gDNA was then normalized to a concentration of 1 ng/µL. Standards were tested against every assay used in the qPCR quantification process. For *S. epidermidis*, *C. acnes*, and *C. striatum*, TaqMan Microbe Detection Assays from ThermoFisher were used, while in-house primer-probes were developed for *S. thermophilus*, *S. hominis*, and *R. dentocariosa* (**Table 2**). The reaction mix and cycling conditions were followed for all assays as per the guidelines provided with the TaqMan™ Fast Advanced Master Mix for qPCR (CAT#: 4444557) with the exception of *S. hominis* assay, which was run at an annealing temperature of 64°C. Cutoffs for CT values were determined as per Hays et al. (Hays et al., 2022) (**Supplementary Figure 1**).

**Table 2:** Commercial (Thermo Fisher) and in-house assays used to amplify unique target sequences for each strain of the consortium.

Strain	Assay ID	Forward Primer	Reverse Primer	Probe
*S. epidermidis*	Ba04646141_s1	—	—	—
*C. acnes*	Ba07922019_s1	—	—	—
*S. thermophilus*	—	CAAGTTTGCACGTGAAGTGCC	CGAACTCACTCGTGAGTTTAAC	GAGTCGTTTGGACGGTGAAGTGTAACTTCG
*S. hominis*	—	GAAGTAACAGTTGAAGATGTTAACAAA	TTCATACCAACAACATCTGATGAT	CTGCTGACGAATCAT
*R. dentocariosa*	—	GTGGTATTCCCCCTCATACAC	CCTTCATAAAGTGCTTATCCATACC	CGTCACGCCGCATCCTACA
*C. striatum*	Ba07921944_s1	—	—	—

### 2.4 Bacterial quantification and relative abundance from microbiome replete tissues

On the day of take down, the SURFACE tissue was removed from the Transwell by gentle scraping to detach the circular disc of skin tissue. It was then processed using Qiagen’s QIAamp DNA Microbiome Kit (Cat #51704) to extract bacterial genomic DNA (henceforth called microbiome gDNA). Briefly, the kit first selectively lyses host cells via a detergent-based method while microbial cells are kept intact. The released host DNA is degraded enzymatically while maintaining the bacterial cells. The bacterial cell lysis is then performed by a mechanical and chemical method for a final isolation of bacterial DNA through a spin-column. Multiple Transwell samples (n≥3) were harvested per condition tested in a single timepoint. Samples were never pooled, a single Transwell tissue’s derived microbial DNA is represented as a single data point.

The abundance of each individual bacterial strain in the microbiome gDNA samples was quantified via qPCR using strain specific assays as listed in **Table 2** and standard curves as shown in **Supplementary Figure S1**. The reaction was run in duplicates for each microbiome gDNA sample and the mean of the duplicates was used to calculate copies for one Transwell tissue.

The Ct value for each strain specific amplification curve was extrapolated to gDNA concentration from the standard curve. The genome size as reported by ATCC was used to calculate the genome copies per picogram of gDNA (**Table 3**). These two values were used to calculate the number of genome copies using the following formula:

**Table 3:** Calculation of approximate genome copies per picogram of extracted DNA.

Strain	Genome Size (bp)	Copies/pg DNA
*C. acnes*	2,497,484	3.653×10**^2^**
*S. thermophilus*	1,861,212	4.902×10**^2^**
*S. hominis*	2,261,062	4.035×10**^2^**
*S. epidermidis*	2,575,951	3.542×10**^2^**
*R. dentocariosa*	2,506,228	3.641×10**^2^**
*C. striatum*	2,945,796	3.097×10**^2^**

Genome sizes were taken from ATCC product information.


$$\begin{array}{c} Number\;of\;genome\;copies\;per\;\mu l\\ =\;gDNA\;concentration\;of\;strain\;from\;microbiome\;\\ sample\;\left(\frac{pg}{\mu l}\right)\times genome\;copies\;per\;pg\;of\;DNA\;\left(\frac{copies}{pg}\right) \end{array}$$

Relative abundance was calculated by summing the genome copies of all strains per microbiome gDNA extract. Biodiversity within groups of samples was measured using Shannon’s diversity index (Shannon, 1948).


$$H=\;-\sum^{\;}[\left(pi\right)x\log\left(pi\right)]\;$$

Where H= Shannon diversity index; pi = proportion of individuals of the -ith species in a whole community or individuals of a given species over total number of individuals in a community.

Ct values from qPCR were analyzed using QuantStudio Real-Time PCR Software v1.7.2 (Thermo Fisher Scientific). Data analysis and plotting was performed using GraphPad Prism software v10.

### 2.5 Histology

On the day of takedown, SURFACE tissues designated for histology were fixed in 4% Paraformaldehyde (Sigma) in Phosphate Buffered Saline (PBS) for at least 15 minutes at room temperature. Tissues were removed from Transwell inserts and stored in 70% ethanol at 4°C for further sectioning and staining steps. All samples were processed at NoVo Vita Histopathology Laboratory (Natick, MA). The staining protocol used was a modified gram stain that was optimized for the detection of Gram-positive and Gram-negative bacteria within tissue as describe in Becerra et al. (Becerra et al., 2016).

### 2.6 RNA isolation and qRT-PCR analysis

Harvested NHEKs from the tissue model were stored at 4°C in RNAlater Stabilization Solution (Thermo Fisher Scientific) until day of RNA extraction. RNA from NHEKs was then isolated using RNeasy Plus Mini Kit (Qiagen) as per vendor instructions.

TaqMan Gene Expression Assays (Thermo Fisher Scientific) were acquired, and target gene expression was normalized using the housekeeping (HK) gene glyceraldehyde-3-phosphate dehydrogenase (GAPDH) (Assay ID: Hs02786624_g1). Gene expression assays for SERPINB4 (Assay ID: Hs00741313_g1) and S100A9 (Assay ID: Hs00610058_m1) were pooled with the HK gene while assays for KRT1 (Assay ID: Hs01549615_g1), FLG (Assay ID: Hs00856927_g1), and DEFB4B (Assay ID: Hs00175474_m1) were run in separate reactions due to large differences in relative transcript levels interfering with parallel amplification.

RNA was isolated from multiple Transwell tissues per condition (n≥3) and reaction was run in duplicates for each RNA sample isolated. Samples were never pooled, single Transwell tissue derived RNA is represented as a single data point. Mean Ct values were used to calculate fold change by delta Ct method over HK gene.

Relative expression of genes of interest were analyzed using QuantStudio Real-Time PCR Software v1.7.2 (Thermo Fisher Scientific). Delta Ct values were plotted, and data analysis, including statistics, was performed using GraphPad Prism software v10.

### 2.7 Luminex

On the day of takedown, media was collected from the basal compartment of each Transwell tissue (n≥3 Transwell tissues per condition). Collected media was filtered through Nanosep centrifugal filters (Cytiva) and frozen at -80°C until day of use. On the day of assay, samples were placed on ice until defrosted. Using a custom Luminex Discovery Assay (R&D Systems), samples were tested for fifteen analytes (TNF-α, IFN-γ, IL-6, IL-8/CXCL-8, IL-1α/IL-1F2, IL-1β/IL-1F2, IL-1ra/IL-1F3, IL-36β/IL-1F8, CCL5/RANTES, CCL20/MIP-3α, CCL27/CTACK, S100A9, TSLP, IL-12p70, CXCL1/GROalpha/KC/CINC-1). Luminex assay was performed following the manufacturer’s instruction and results were read using the FlexMap 3D program (FlexMAP). Data was analyzed and exported using FlexMAP 3D Xponent software. Standard curve results were verified and fit to five- or four-point log weighted scales ensuring all R^2^ >.99. Exported data was normalized to account for 1:2 dilution during assay. Final values were graphed and analyzed for statistical significance using GraphPad Prism software v10.

## 3 Results

### 3.1 Skin-commensal co-culture method development

**Figure 1:**
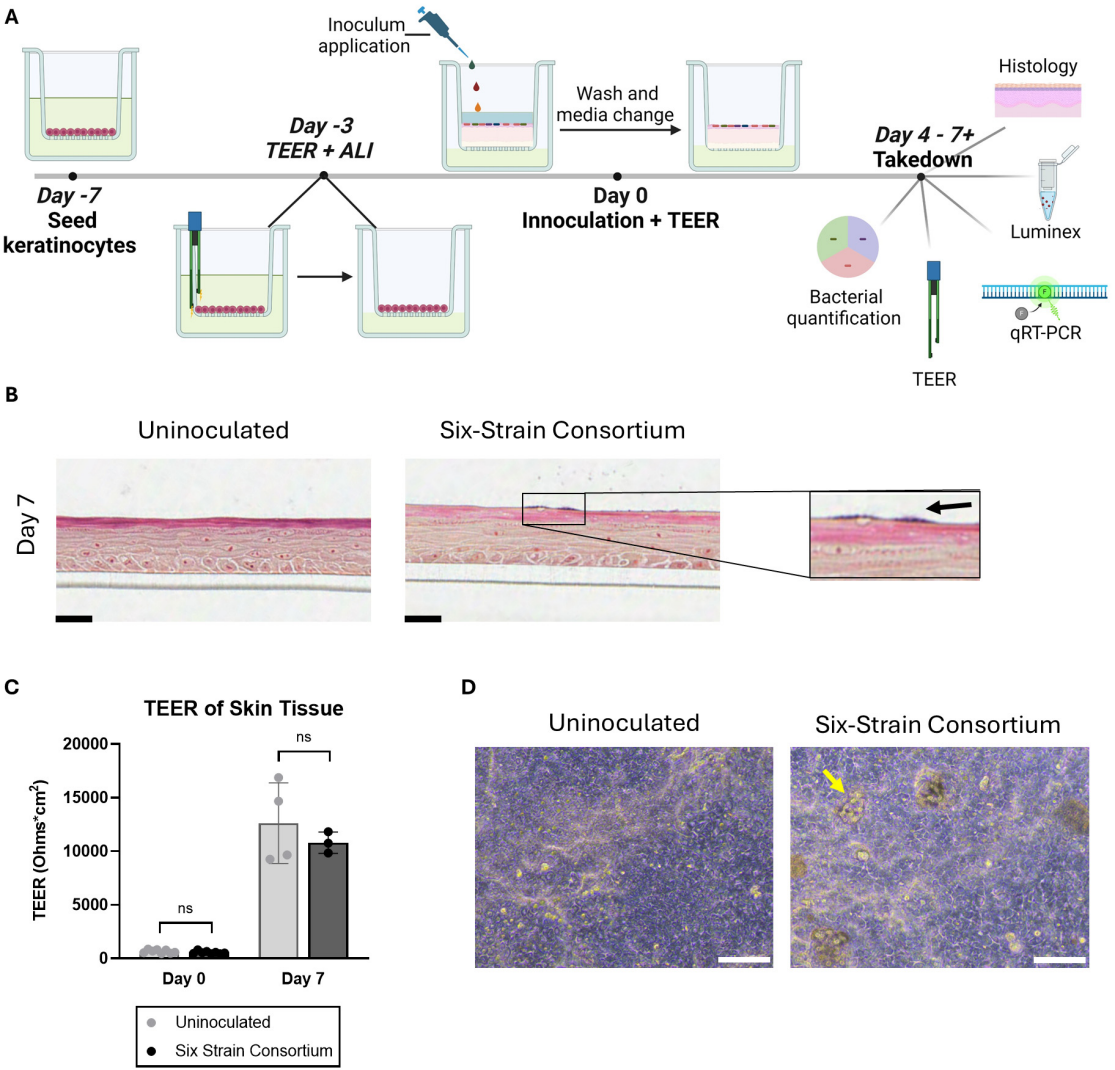
Overview of the *in vitro* model of a human skin microbiome, SURFACE. **(A)** Experimental timeline of SURFACE establishment, including cell seeding, air-liquid interface (ALI) introduction, inoculation of 6-strain consortium, and model readouts. Illustration was drawn with Biorender software. **(B)** Histological sections of tissue with and without a 6-strain microbiome at days 4- and 7- post-inoculation. Tissue was stained with modified hematoxylin and eosin Gram stain with a safranin counterstain. Scale bars represent 25µm. **(C)** Tissue barrier function measured by TEER at day 0 (before inoculation) and 7 days post-inoculation as compared to uninoculated tissue. Each dot represents TEER measured on a single Transwell tissue which was uninoculated (grey dots) or inoculated with the six-strain consortium (black dots). TEER was not measured on one instance of bacterial overgrowth and subsequent barrier breach observed on Day 7 in an inoculated tissue sample. Significance was determined by Multiple unpaired t-test with Welch correction, p<.05. **(D)** Phase contrast images of the differentiated SURFACE tissue in uninoculated conditions (left) and with resident six-strain consortium (right) on Days 4 post-inoculation. Yellow arrows point to an example bacterial colony on the surface of the tissue. Scale bar represents 100 µm. Ns, not significant.

Three main parameters were identified as critical to the establishment of the SURFACE model: barrier formation by NHEKs prior to bacterial introduction, microbial composition of inoculum, and inoculum titration. An epidermal-equivalent tissue with relevant barrier function was grown from NHEKs over the two weeks of total culture time and is described in **Figure 1**. Total culture time included initial NHEK seeding at Day -7, introduction of ALI at Day -3, inoculation at Day 0, and 7 days of co-culture with the 6-strain consortium, at which point samples were collected for various assays (**Figure 1A**). Prior to multi-strain microbial inoculation, NHEKs established TEER (Trans-epithelial electrical resistance) of at least 300 Ohms*cm^2^, providing an impermeable surface on which to seed the commensal microbes.

Strains of human skin commensal bacteria were selected based on three main criteria: prevalence in previous literature and the Human Microbiome Project (Turnbaugh et al., 2007), availability of the strain from commercial vendors so that the strain source and genome are well documented, and diversity of strains at the genus level to allow for microbial quantification using a strain-specific assay. The final consortium comprises *S. epidermidis*, *C. acnes*, *S. thermophilus*, *S. hominis*, *R. dentocariosa*, and *C. striatum*. We adopted an optical density-based method to produce a consistent inoculum at the strain level. This approach works around two challenges encountered in direct enumeration of the applied consortium. First, individual species of the consortium require specific growth supplements and atmospheric conditions disallowing enumeration after mixing. Natural resistance to antibiotics was determined with standard e-test (data not shown), however no single strain was uniquely resistant to a specific antibiotic precluding enumeration in antibiotic selective agar plates. Second, CFU counts are not available at the time of consortium inoculation due to multi-day growth conditions. We therefore titrated each strain to a measured OD and diluted from this to generate components of the consortium. After screening multiple dilutions of specific strains, we identified an optimal dilution factor for each strain in the consortium that allowed for prolonged tissue culture but avoided bacterial overgrowth. Prior to mixing the consortium strains for inoculation, the diluted strains were plated in appropriate agar plates (**Table 1**) for CFU enumeration. This served as a benchmark of applied strains across experiments. The composition of the applied inoculum varied between experiments despite similar preparation (**Figurea 2A**; **Supplementary Figure S2A**), however this variability was tolerated by our model across experiments where microbiome replete tissue continued to show rise in TEER (**Figure 1C**; **Supplementary Figures S2C**, **S3C**).

**Figure 2:**
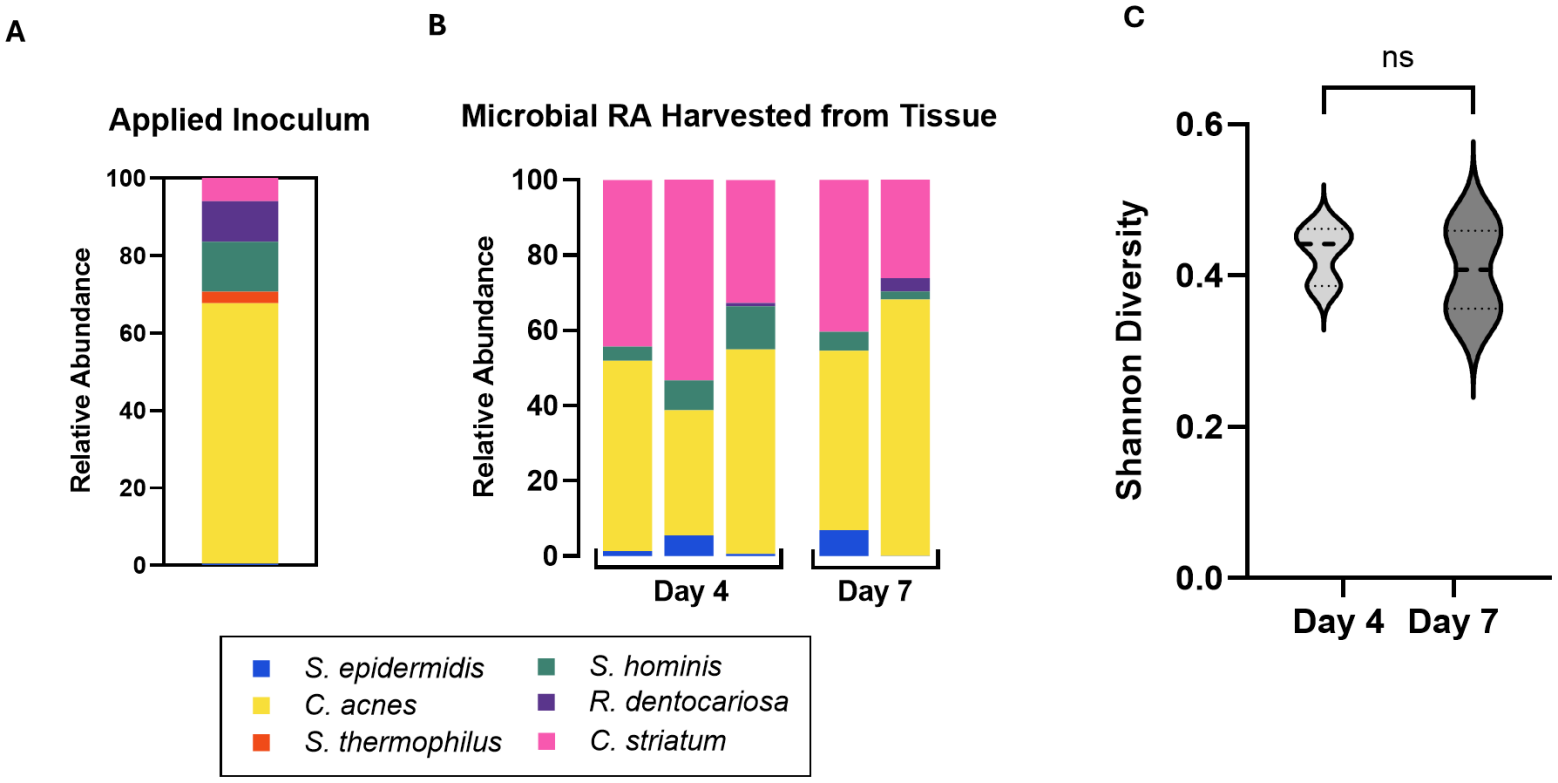
**(A)** Relative abundance of the six applied consortia species in applied inoculum on Day 0 as measured by CFU. **(B)** Relative Abundance of the six strains harvested from skin tissue on Day 4 and Day 7 as measured by qPCR of microbiome genomic DNA. Absolute abundance values are reported in **Table 4**. Each bar represents a single Transwell tissue within an experiment. **(C)** Alpha diversity was interpreted using Shannon Diversity. Significance was determined by unpaired t-test with Welch correction. Ns, not significant.

#### 3.1.1 Six strain consortium co-culture on skin tissue

Following introduction of bacteria, NHEKs maintained characteristic tissue structure on days 4 and 7 post-inoculation, with spatially distinct apical and basal layers (**Figure 1B**). The skin barrier, as measured by TEER, increased ~10 fold from time of inoculation to time of take-down 7 days later. On the first day of inoculation, Day 0, the TEER average was ~600 Ohms*cm^2^, while on Day 7 average TEER reached over 12,000 Ohms*cm^2^ (**Figure 1C**). A similar trend in barrier function was seen independent of microbes presence, indicating that the tissue continued to improve barrier function after the bacteria were co-cultured on the skin surface.

Initial trial studies tested up to 4 days of microbiome integration, at which point bacterial relative abundance (**Supplementary Figure S2B**) and TEER (**Supplementary Figure S2C**) were measured. Subsequent studies were extended out to 7 days after microbiome addition. Each figure corresponds to a single experiment in which at least 2 Transwells replicates were allocated per condition.

Occasionally, tissues were damaged during handling, resulting in microbial access to the lower chamber, overgrowth in the rich media and ultimately tissue breakdown; in these cases, it was appropriate to identify and exclude the individual samples from the data set. Indeed, barrier breach instances decreased when tissue handling procedures were modified to minimize touch time on the Transwell.

Histological sectioning of the tissue demonstrated the complex tissue architecture including layers similar to *in vivo* basal, spinous, granulosum and stratum corneum layers (**Figure 1B**) (Yousef et al., 2024). Evidence of early cornification is seen in tissues as a pink apical layer. A modified Gram stain (Becerra et al., 2016) highlights in dark purple the presence of bacterial colonies on the surface of the skin, which appear in isolated groups and are mirrored by phase contrast images of the tissue (**Figures 1B, D**). In summary, the SURFACE model shows stable skin tissue characteristics such as high TEER and stratified morphology, providing a relevant testbed for human skin microbiome studies.

#### 3.1.2 Microbial consortium characterization

To characterize the stability of the six-strain microbial consortium on our tissue model, we determined the relative genetic abundance of the applied strains over the course of 7 days. On day 0, inoculum containing all six strains in the ratios established (as above) was applied to the surface of each skin tissue sample. The applied inoculum was enumerated by CFU plating the individual strains used for creating the mixed inoculum (**Figure 2A**) indicating viability of the strains. Once the strains were combined and cultured together on the tissue model, it was not possible to measure CFU of individual strains, given differential culture conditions for each strain. Therefore, on Day 4 and Day 7 following inoculation of the six-strain consortium, the resulting microbial composition on the tissue was evaluated using qPCR analysis of strain-specific target sequences to enumerate genome copies. Specificity of each strain-specific PCR assay was evaluated for cross-reactivity with the other strains (**Supplementary Figure S1A**). To calculate absolute copies of each strain present we used a two-step method to extrapolate genomic DNA to genome copies. First, standard curves were generated from known concentrations of genomic DNA of each strain to support the conversion of Ct value to absolute genomic DNA (**Supplementary Figure S1B**). Then we determined the absolute weight of a single genome to extrapolate the number of genomic copies present in strain specific genomic DNA yielded from the microbiome sample (**Table 3**).

Using these tools, absolute genome copies were measured from total extracted microbial genomic DNA on days 4 and 7 post-inoculation of the skin tissue as shown in **Table 4** and plotted as relative abundance (**Figure 2B**). On day 4, genome copies of *C. acnes* and *C. striatum* were dominant on the tissue. *S. epidermidis, S. hominis*, and *R. dentocariosa* were present at low but detectable levels (**Figure 2B**; **Table 4**). *S. thermophilus* genome was undetectable. By day 7 of culture with the six-strain consortium, the skin surface was still supporting five of the six bacterial strains and remained relatively stable based on calculated relative genetic abundance. Furthermore, the alpha diversity of the strains - using Shannon diversity index - illustrates no significant shift in alpha diversity between days 4 and 7 of co-culture (**Figure 2C**). The shift to a stable state with high representation of *C. acnes* and *C. striatum* was reproduced over two independent experiments (**Supplementary Figure S2B**). Although *S. thermophilus* was no longer detected by day 4, its exclusion from the inoculum in prior studies led to decreased barrier function and increased barrier breach incidence. When *S. thermophilus* was excluded from the consortium, 30% (3/10) tissue replicates experienced barrier breach by 7 days post-inoculation, while no tissues that included *S. thermophilus* lost barrier function (0/14) (data not shown).

**Table 4:** Calculated total genome copies per Transwell (0.33cm^2^) represented in **Figure 2B**.

	*S. epidermidis*	*C. acnes*	*S. thermophilus*	*S. hominis*	*R. dentocariosa*	*C. striatum*	Total Genome Copies
**Day 4**	6.72E+04	2.43E+06	0.00E+00	1.82E+05	0.00E+00	2.13E+06	*4.80E+06*
	2.91E+05	1.73E+06	0.00E+00	4.12E+05	0.00E+00	2.77E+06	*5.21E+06*
	2.09E+04	1.82E+06	0.00E+00	3.87E+05	2.67E+04	1.10E+06	*3.35E+06*
**Day 7**	7.57E+05	5.20E+06	0.00E+00	5.46E+05	5.30E+03	4.41E+06	*1.09E+07*
	9.79E+03	5.05E+06	0.00E+00	1.60E+05	2.57E+05	1.94E+06	*7.41E+06*

Total tissue was collected on the indicated days post-inoculation. Values in each row represents genome copies from a single Transwell tissue. Genome copies were calculated using the formula in Section 2.4.

Several strains in our six-strain consortium require specific media supplements or atmospheric conditions for growth in defined media (**Table 1**). These constraints precluded co-culture of strains under standard growth conditions to study species-level interactions. This is demonstrated when the six-strain inoculum was plated in typical growth conditions, Tryptic Soy Agar, under aerobic conditions for 24 hours- the resulting colonies were entirely *S. hominis* (**Supplementary Figure S4**). The same initial inoculum of our core consortia strains when applied on the *in vitro* skin model was able to support an assortment of strains in one test bed (**Figure 2B**; **Supplementary Figure S4B**), including the anaerobic strain, *Cutibacterium acnes*. These findings indicate that the skin surface provides a more supportive growth environment for commensals compared to agar, enabling the simultaneous existence of at least 5 strains in one microenvironment.

### 3.2 Effect of commensal microbes on a model of atopic dermatitis

**Figure 3:**
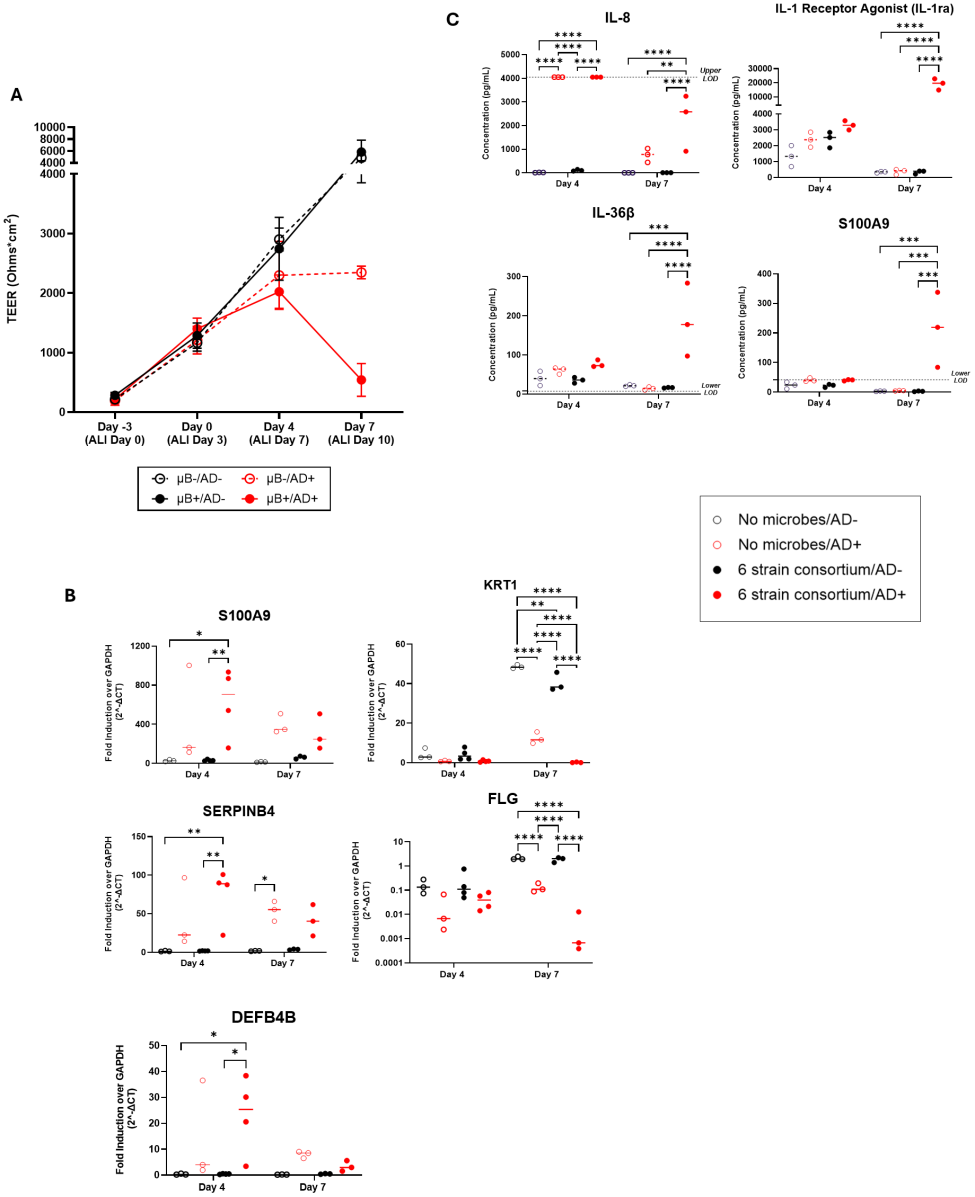
**(A)** Measured TEER of tissue with (filled circle) or without (open circle) bacterial consortium inoculation and with (red circle) or without (black circle) simulated AD-cytokine mix added to basal media. **(B)** S100A9, SERPINB4, DEFB4B, KRT1, and FLG gene expression in tissues with cytokine mix added to the basal media (AD+) compared to tissues without the mixture (AD-) on the skin model with or without bacterial consortium. **(C)** Levels (pg/mL) of IL-8/CXCL8, S100A9, IL-1ra/IL-1F3, and IL-36β secretion in the basal media as determined by Luminex. Dotted horizontal line represents upper limit of detection (IL-8) or blank media control (S100A9, IL-36β). Each dot represents data collected from a single Transwell tissue within an experiment. Statistical significance was determined using Two-way ANOVA with Tukey’s multiple comparisons. Any comparisons not shown are non-significant. *p<0.05, **p<0.01, ***p<0.001 and ****p<0.0001. Ns, not significant.

We wished to expand the capability of the SURFACE model by studying the effect of a relevant skin disease on the tissue testbed. At the time of bacterial inoculation, a pro-inflammatory cytokine mixture of IL-22, TNF-α, IL-4, and IL-13 was added to the basal media to simulate atopic dermatitis (AD+) while healthy control conditions were maintained in standard growth media (AD-). Through the seven days of co-culture, the AD- tissue continued to rise in TEER regardless of the presence of the microbiome. In AD+ tissues, TEER increased from Day 0 to Day 4 in the absence of the microbiome and plateaued by day 7. However, in the presence of the microbiome, TEER declined, suggesting an additive effect on loss of barrier function, but without observable spread of bacteria into the basal media (**Figure 3A**; **Supplementary Figure S3C**).

To better understand the mechanism of tissue breakdown, we evaluated host tissue gene expression of 5 literature-derived AD-associated markers at day 4 and day 7 using q-RT PCR (Sivaprasad et al., 2015; Totsuka et al., 2017; Nedoszytko et al., 2020; Lang et al., 2021). S100A9, SERPINB4, DEFB4B, KRT1, and FLG were found to be differentially expressed in tissues exposed to AD+ conditions (**Figure 3B**). At day 4 post-inoculation, there was significant upregulation of the genes S100A9, SERPINB4, and DEFB4B in inoculated AD+ tissue when compared to inoculated AD- tissue (**Figure 3B**). No significant increase in these genes was measured as a result of introduction of only bacteria or from AD cytokines alone, indicating a synergistic response to the combination of bacteria and cytokines. At day 7 post-inoculation, all three genes for secreted proteins trended to higher expression with cytokine exposure irrespective of inoculation, although only reached significance in the case of SERPINB4 without bacteria. Structural genes KRT1 and FLG were down regulated in the AD+ condition, and this effect was not observed until day 7 post-inoculation (**Figure 3B**). The KRT1 downregulation was observed when disease cytokines or microbes were introduced independent of each other. This was further exacerbated by introduction of both perturbations. FLG downregulation was significantly impacted by disease but the addition of microbes had no significant changes in gene expression although a downward trend was observed.

In parallel, we collected media and evaluated secreted factors with a focus on cytokines and secreted factors predominantly expressed by keratinocytes: IL-8, IL-1β, IL-1ra, CCL20, S100A9, TSLP, IL-36β, CCL27, CXCL1, TNFα, IL-6, IFNγ, CCL5, and IL-12 p70. Luminex assays were performed on the basal media collected at days 4 and 7. Out of the 15 selected analytes (**Supplementary Table S1**), four showed notable trends in diseased condition with or without microbiome presence (**Figure 3C**). On day 4, IL-8 was significantly upregulated in the diseased conditions irrespective of bacteria addition. The levels of IL-8 declined by day 7, at which point levels were significantly higher in only in the disease plus microbe context compared to either perturbation alone. S100A9, IL-36β and IL-1 Receptor Antagonist (IL-1RA) were also significantly changed across the experiment; in these cases, no significant change was measured on day 4, but on day 7 the combination of disease-driving cytokines plus microbes uniquely induced significant secretion of the factors (**Figure 3C**). Overall, a synergistic effect of the AD+ cytokines with commensal bacteria can drive secretion of cytokines from NHEKs after seven days of co-culture. Overall, the SURFACE model responds to AD-associated cytokines with relevant tissue responses, and the integration of commensal bacteria sensitizes the tissue to disease induction.

### 3.3 Induction of dysbiosis in the disease model

**Figure 4:**
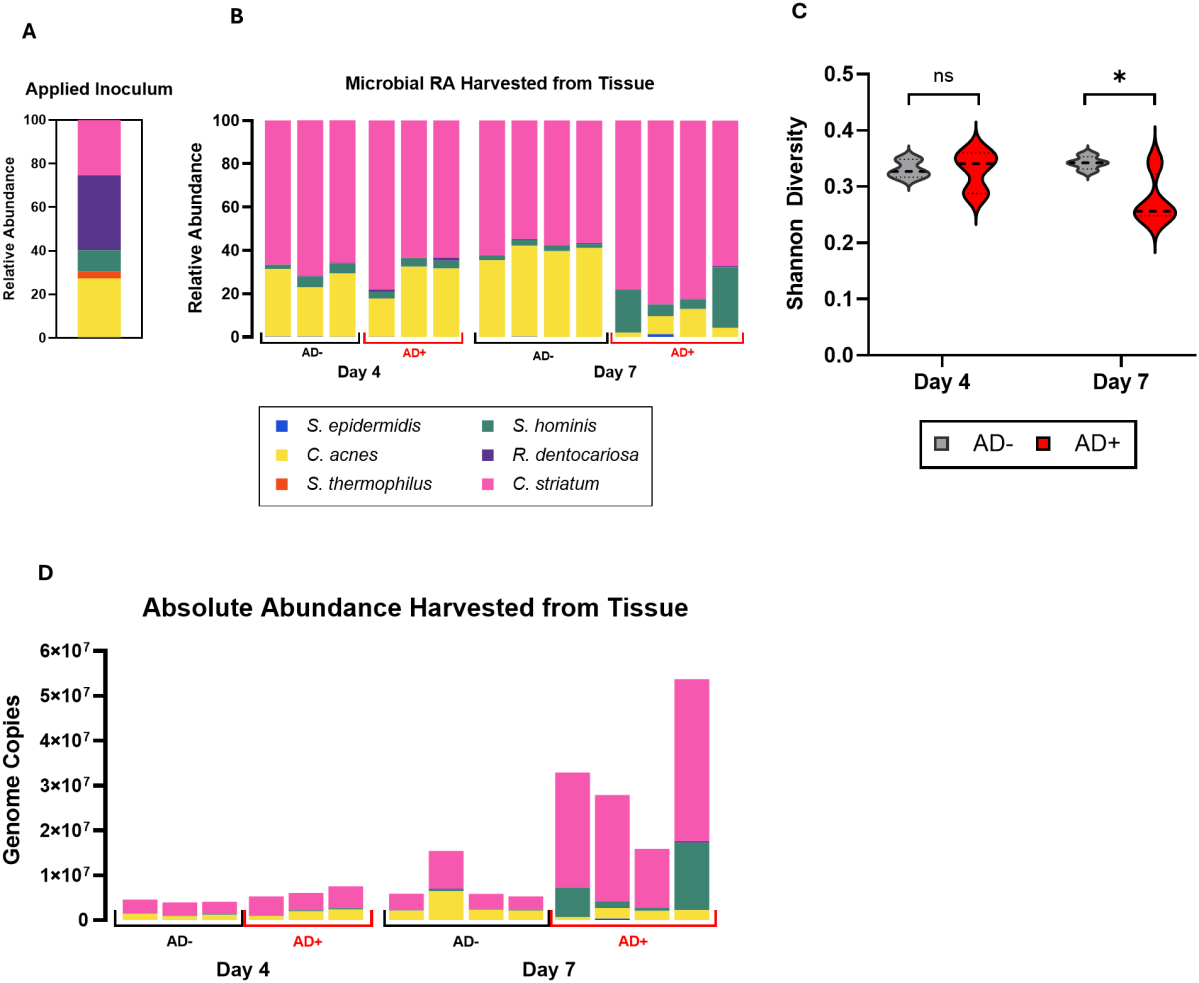
**(A)** Relative abundance of applied inoculum on Day 0 as measured by CFU. **(B)** Relative abundance of the six strains on Day 4 and Day 7 as measured by strain specific qPCR assays of microbiome genomic DNA extracted from tissues with (AD+) or without (AD-) the addition of an AD cytokine mix. **(C)** Alpha Diversity of relative abundance as interpreted by a Shannon Diversity calculation. **(D)** Absolute abundance of strains harvested from tissue as measured by qPCR. Each bar represents microbiome harvested from single Transwell tissue within an experiment. Numerical values of absolute abundance are reported in **Table 5**. Significance was determined by Multiple unpaired t-test with Welch correction, *p<.05. Ns, not significant.

In addition to the changes to the host epithelium, the introduction of AD+ cytokines impacted the consortium of microbes present on the skin model. Notably, the modulation of the consortium was not observed until 7 days after inoculation. Two groups of skin tissue samples were treated with inoculum of identical composition (**Figure 4A**) and grown in identical conditions aside from the introduction of the disease state by concurrent addition of the AD+ cytokines. At day 4 post-inoculation and introduction of AD+ conditions, there was not a statistically significant difference in consortia composition detected between the two groups (**Figure 4C**). However, there was a slight increase in absolute bacterial abundance in the AD+ group compared to the untreated group (p= 0.0385, Student’s t-test) (**Figure 4D**; **Table 5**). By day 7, however, there was a marked change in the composition between the AD+ and AD- groups. While the untreated tissues maintained a similar consortia composition to the previous timepoint, the experimental AD+ group showed decreased abundance of *C. acnes* and increased *C. striatum* and *S. hominis*, with an overall significant decrease in Shannon diversity (**Figure 4B, C**). In addition, there was an increase in overall microbial abundance under AD+ conditions (p=0.0252, Student’s t-test) (**Figure 4D**; **Table 5**). The dysbiosis was observed across a total of 2 experiments with Shannon Diversity trending downward (**Supplementary Figures S3A**, **S3B**). Our findings indicate that SURFACE provides sufficient complexity in the microbiome to support evaluation of changes to composition and abundance.

**Table 5:** Calculated total genome copies per Transwell (0.33 cm^2^) represented in **Figures 4B, D**.

		*S. epidermidis*	*C. acnes*	*S. thermophilus*	*S. hominis*	*R. dentocariosa*	*C. striatum*	Total Genome Copies
**Day 4**	**AD-**	9.30E+03	1.44E+06	0.00E+00	8.64E+04	5.33E+03	3.07E+06	*4.61E+06*
		9.29E+03	9.01E+05	0.00E+00	1.96E+05	1.03E+04	2.84E+06	*3.96E+06*
		5.39E+03	1.20E+06	0.00E+00	1.92E+05	1.06E+04	2.70E+06	*4.11E+06*
	**AD+**	7.57E+03	9.43E+05	0.00E+00	1.69E+05	5.04E+04	4.16E+06	*5.33E+06*
		0.00E+00	1.97E+06	0.00E+00	2.45E+05	0.00E+00	3.84E+06	*6.06E+06*
		0.00E+00	2.40E+06	0.00E+00	2.74E+05	9.50E+04	4.80E+06	*7.58E+06*
**Day 7**	**AD-**	4.87E+03	2.10E+06	0.00E+00	1.37E+05	0.00E+00	3.69E+06	*5.93E+06*
		2.62E+04	6.51E+06	0.00E+00	4.13E+05	5.55E+04	8.49E+06	*1.55E+07*
		4.06E+03	2.33E+06	0.00E+00	1.63E+05	0.00E+00	3.38E+06	*5.87E+06*
		0.00E+00	2.18E+06	0.00E+00	1.03E+05	2.14E+04	3.00E+06	*5.31E+06*
	**AD+**	0.00E+00	6.86E+05	0.00E+00	6.53E+06	9.56E+03	2.57E+07	*3.29E+07*
		3.72E+05	2.35E+06	0.00E+00	1.50E+06	2.25E+04	2.37E+07	*2.80E+07*
		3.83E+03	2.06E+06	0.00E+00	7.02E+05	4.75E+04	1.31E+07	*1.59E+07*
		0.00E+00	2.26E+06	0.00E+00	1.50E+07	3.53E+05	3.61E+07	*5.37E+07*

Total tissue was collected on the indicated days post-inoculation and in the presence (+) or absence (–) of added cytokines (AD). Values in each row represent genome copies from a single Transwell tissue. Genome copies were calculated using the formula in section 2.4.

## 4 Discussion

### 4.1 A novel model of host-microbe interaction

The human skin microbiome model reported here represents a major advance in the complexity and longevity of *in vitro* models containing both host tissue and a microbial consortium. There have been several studies reporting 3D-Skin tissue models as ideal substrates to study strain-strain interactions or the establishment of stable microbiome for durations ranging from hours to a few days (Larson et al., 2021; Holland et al., 2008; Holland et al., 2008; Kirker et al., 2009; de Breij et al., 2012; Haisma et al., 2013; Popov et al., 2014; Cadau et al., 2017; Lemoine et al., 2020; Kohda et al., 2021; Lemoine et al., 2021; Loomis et al., 2021). In our current work, we have demonstrated the feasibility of establishing a stable microbiome on a skin tissue model with six important skin microbiome strains for a period of 7 days. Furthermore, we showed the potential to study chronic skin diseases to understand the interaction between host and microbe during pathogenesis. This body of work is the first of its kind to demonstrate consistent modulation of the microbiome in response to a disease state in a physiologically relevant *in vitro* model of human skin microbiome.

A review from Smyth and Wilkinson comprehensively summarizes the challenges associated with skin microbiome research (Smythe and Wilkinson, 2023). Microbiome research relies largely on sample collection from healthy or diseased volunteers and longitudinal analysis of collected samples by 16s, shotgun or whole genome sequencing (Chng et al., 2016). For instance, Kang et al. were able to uncover the molecular mechanism driving Acne in humans supplemented with vitamin B12 via meta transcriptomic profile of microbes in Acne patients vs normal individuals (Kang et al., 2015). Zhang et al. performed elegant studies to demonstrate via mouse models and extracts from skin of healthy vs Dengue and Zika virus-infected patients that flavivirus promoted the growth of acetophenone producing bacteria in the microbiome (Zhang et al., 2022). While these methods are helpful in hypothesis-generating research to understand correlative factors in microbiome and disease states, they often cannot uncover cause vs. effect of dysbiosis or inflammation. Such characterization will be well served by *in vitro* models that capture microbial metabolism in the context of a diverse microbial population colonizing the skin. To provide a relevant, skin-like microenvironment for microbial culture, we cultured NHEKs for 7 days including at ALI, generating a complex, differentiated skin tissue including a layer of cornified epithelium on the surface providing a potentially relevant microenvironment for skin microbiome culture. At this point, the bacterial consortium was added and co-cultured for an additional 7 days. At day 7 the tissues remained stable with six-strain consortium suggesting we could extend duration to >7 days. Further prolonged culture periods may define a steady state of microbial growth such that the total number of bacteria are in equilibrium with the tissue culture.

The establishment of a multi-strain, multi-day skin microbiome model depends on the initial concentration of single strains as well as the ratios between consortium strains. However, there is an innate variability of starting inoculum that is due to titrating small volumes and numbers of total bacteria, which can be seen across up to 3 experiments in the day 0 CFU enumeration. Despite the Day 0 inoculum variability, a multi-strain consortium of human skin microbes is still supported on skin tissue for 4-7 days, indicating a degree of robustness to the protocol, and emphasizing aspects that are likely critical to model establishment: strain type, relative strain amounts and inoculation methodology.

Characterization of consortium composition was critical to our understanding of the model over time. However, characterization of a mixed population of bacteria is not straightforward given the range of genera involved and their variety of selective culture conditions. Therefore, we chose to work with strains for which unique PCR-based assays were commercially available or could be designed. This allowed for the calculation of relative microbial abundance across multiple Transwell replicates, although assumptions were made. First, we assumed a single genome copy per bacterial cell, which may not be the case in actively dividing bacterial cells. Confidence in this assumption was provided from our characterization that the rate of bacterial division was slow based on the total microbial genomic DNA yield on day 7 which was less than twice the yield from day 4. Literature also supports that commensal microbes on the skin are slow to replicate compared to the denser gut microbiome (Lu et al., 2014; Byrd et al., 2018; Cundell, 2018). Second, we are aware that any persistent plasmid DNA in a bacterial strain would add to the total DNA weight extracted; this genetic material is not accounted for in genome size-based calculations. For these calculations, we assumed that this DNA did not contribute significantly to our overall composition calculations.

Estimates of bacterial density in the human skin vary depending on the method of collection (tape lift, punch biopsy) and subsequent culture methods making it challenging to interrogate the full range of microbes present. When approximated, the total concentration of bacterial genomes normalized to the surface area in our model ranges from 1E+7/cm^2^ to 3E+7/cm^2^ (**Table 4**). This density of bacterial cells is >10-fold higher than 1E+3 to 1E+5 in most skin areas as measured by a more stringent CFU method for aerobic bacteria (Reichel et al., 2011). The genetic method used here, which captures any residual genomic sequence from dead bacterial cells, would be expected to overestimate the number of viable cells/area.

Of the six strains introduced to the skin tissue on day 0, five species are present in the final stable population across both timepoints investigated. *S. thermophilus* fails to appear in detectable quantities through qPCR analysis across multiple timepoints, Transwell replicates, and experimental trials. Despite this, the addition of *S. thermophilus* appears to impact the overall health and composition of the model and therefore was maintained as a member of the consortia used in these studies. This finding indicates the potential role that *S. thermophilus* may play in the early establishment of a commensal relationship within the microbiota and the host tissue. *S. thermophilus* is widely used as a prebiotic agent and has been shown to increase ceramide levels in stratum corneum (Di Marzio et al., 2003; Lombardi et al., 2022). We speculate that its presence early in coculture may provide a metabolic niche that supports establishing the surviving strains as a balanced consortium.

### 4.2 Disease modeling

A key facet of an *in vitro* skin tissue culture-based model is to mimic skin conditions such as atopic dermatitis, psoriasis, pathogen response, and wound healing. Integration of the microbiome on healthy or diseased skin enables us to probe underlying mechanisms and therapeutic interventions. The host immune system is a key modulator of microbiome balance as well as a primary host response to pathogens and dysbiosis (Belkaid and Harrison, 2017). To capture inflammatory responses associated with chronic skin diseases we adopted a previously established acellular model of atopic dermatitis in which NHEK are exposed to a cytokine milieu that is characteristic of AD (Bernard et al., 2012). Previous work showed upregulation of AD associated biomarkers but the effect of the microbiome was not explored. In this work, we significantly expanded the characterization of the host tissue response to these cytokines and evaluated changes to our bacterial consortium as the result of epithelial damage induction. Simulated AD disease states were apparent, although not until Day 7, based on tissue gene expression of key AD associated markers and shifts in microbial composition, indicating the critical need for long term culture systems. Our model provides necessary longitudinal growth to support characterization of disease states and microbial response that happens over extended timelines.

Currently, AD pathology is primarily determined by epithelial barrier function and immune cell response (Bin and Leung, 2016). In our study involving disease induction, we were particularly interested to characterize the NHEK response as proof of relevant disease induction, and to expand characterization of secreted factors that could affect microbiome balance. S100A9 and S100A8 belong to the Damage Associated Molecular Pattern (DAMP) family of secreted factors and are elevated in AD patient serum (Saito-Sasaki and Sawada, 2023; Guttman-Yassky et al., 2024). SERPINB4 and SERPINB3 are serine protease inhibitors which are elevated in chronic inflammatory diseases and serve an important role in the cross-linking of structural proteins, moderating immune responses, and maintaining of epidermal homeostasis (Sun et al., 2017). In clinical atopic dermatitis, expression of SERPINB3/B4 is thought to be induced by Th2 inflammatory cytokines, IL-4 and IL-13 (Sun et al., 2017). DEFB4b is a beta-defensin produced by activated epithelial cells and exhibits high levels of antimicrobial activity towards Gram-negative bacteria (García et al., 2001; Sharma and Nagaraj, 2015; Meade and O’Farrelly, 2019). It is significantly overexpressed in AD tissue and other inflammatory skin conditions (Cieślik et al., 2021). At the gene expression level, S100A9, SERPINB4 and DEFB4B were all upregulated in the NHEK tissue at 4 days following introduction of cytokines, but only significantly increased in the presence of bacteria. By 7 days following disease induction, upregulated gene expression subsided to non-significance over healthy tissue. In a similar but delayed pattern, key secreted proteins were upregulated by day 7 following cytokine introduction, but only in the presence of consortia. IL-8, IL-36β and IL-1ra factors are produced by keratinocytes in response to inflammatory factors, although they play distinct pro- and anti-inflammatory roles (Iznardo and Puig, 2022). Blockade of IL-1 family factors is being researched as a therapeutic strategy for treating AD (Dinarello et al., 2012). IL-8 was strongly secreted by day 4 in response to disease induction, but by day 7 modulated with significantly higher detection only in the presence of consortium. It is possible that bacteria sensitize cells to upregulate these genes or change the secretion of the proteins. In the singular case of S100A9 where we were able to evaluate both gene expression and protein secretion, gene expression parallels protein secretion. The synergistic induction of relevant AD genes and proteins suggests that bacteria can sensitize the tissue response to inflammatory cues and demonstrates the critical importance of including relevant microbial species in tissue models when studying human disease mechanisms.

Filaggrin (FLG) is an important epidermal structural protein required for corneocyte formation, production of water retention molecules, and maintenance of pH stability within the stratum corneum (Totsuka et al., 2017). FLG deficiency is correlated with keratinocyte cellular abnormalities and FLG gene mutations are a high-risk indicator of AD pathogenesis (Osawa et al., 2011; Gupta and Margolis, 2020). Keratin 1 (KRT1) is a marker gene for early differentiation of tissue, and an integral component of the intermediate filament cytoskeleton, providing structural integrity to keratinocytes (Roth et al., 2012). One study demonstrated that KRT and other structural proteins and adhesion molecules were downregulated in the context of AD lesional skin, impairing tissue barrier function (Totsuka et al., 2017). Upon exposure to the AD cytokine cocktail, we measured a significant decrease in KRT1 and FLG transcripts, a decrease that was present with and without bacteria. However, the decrease in both genes was significantly enhanced in the presence of bacteria. This result accurately matches the observed effects on TEER, where bacteria alone did not change tissue integrity, while the effect of AD cytokines was significantly and synergistically enhanced by addition of bacteria. Taken together, the data indicate that the inclusion of commensal bacterial enhances the sensitivity of human skin tissue to inflammatory factors, recommending the inclusion of microbes in mechanistic studies of human skin disease.

### 4.3 Microbiome shifts in disease context

Chronic disease phenotypes like atopic dermatitis and psoriasis are characterized by periods of flare up and punctuated by changes in microbial diversity (Grice et al., 2009; Chng et al., 2016; Fyhrquist et al., 2019; Edslev et al., 2020). AD patients are characterized by distinct microbial signatures and general decline in microbial diversity with dominance of some species such as *S. aureus* and depletion of *Dermacoccus* spp (Tay et al., 2021; Koh et al., 2022). Reduction in *C. acnes* abundance in AD diseased tissue has been previously reported in human sample collection studies (Fyhrquist et al., 2019; Rozas et al., 2021; Green et al., 2023). Furthermore, reinvestigation of metagenomic data sets from pediatric AD cohorts revealed increases in *Corynebacterium kefirresidentii* during and after AD flares (Salamzade et al., 2022). All of these studies rely on human sampling-based approaches, which do not allow prospective studies of cause and effect during pathogenesis. Our model provides a test bed to study host-microbiome mechanisms that drive disease states characterized by dysbiosis and inflammation. This work demonstrates a reproducible change in microbial balance in response to a disease state, where the combination of bacteria outgrowth and loss of diversity mirrors the human disease. The ability to mimic aspects of microbiome response highlights a strength of this system. By integrating a defined but complex consortium of bacteria, we are able to measure variations in the composition. More complex readouts across a range of multi-omic approaches will provide a clearer understanding of the factors that drive the dysbiosis, while alterations in timing of addition of disease and microbes will enhance cause and effect understanding. In addition, there is an opportunity to evaluate the role of pathobionts such as *S. aureus*, a species strongly indicated as a driving factor in AD (Seite et al., 2014). Preliminary work with *S. aureus* in this model resulted in the strain rapidly taking over the culture and immediate termination of the studies (data not shown). Modified strategies for introduction of the species will allow us to study its causative role in disease initiation. Further, this model provides a basis to evaluate the role of the commensal microbiome in suppression or enhancement of pathogenesis from exogenous bacteria and viruses. The demonstration that the microbial consortium provides a consistent and relevant response to introduction of a disease state presents the most robust platform that we are aware of for controlled study of the symbiotic connection between microbial community and host biology.

Taken together, the data presented here demonstrate a first of its kind platform that provides an *in vitro*, human-specific skin model system of the host tissue and defined microbiome. We have highlighted the importance of including relevant microbes when studying inflammatory disease by showing enhanced sensitivity of the tissue to cytokines in the presence of commensal bacterial species, particularly after 7 days of co-culture. In addition, we have shown the utility of the platform for characterizing the responsiveness of the microbiome to host biology and indicated the potential to use these methods to better understand symbiotic relationships among bacterial strains. We recognize significant potential to leverage this type of model system to provide new understanding of host-microbe symbiosis, to characterize safety of skin applications, and to unlock potential therapeutic opportunities present in the skin microbiome.

## Data Availability

The original contributions presented in the study are included in the article/**Supplementary Material**. Further inquiries can be directed to the corresponding author.
