# Supplementary figures and images for: A multi-strain human skin microbiome model provides a testbed for disease modeling
Source: Front Microbiomes. 2025 Feb 4;4:1473292. doi: 10.3389/frmbi.2025.1473292 (PMC12993502; doi:10.3389/frmbi.2025.1473292)

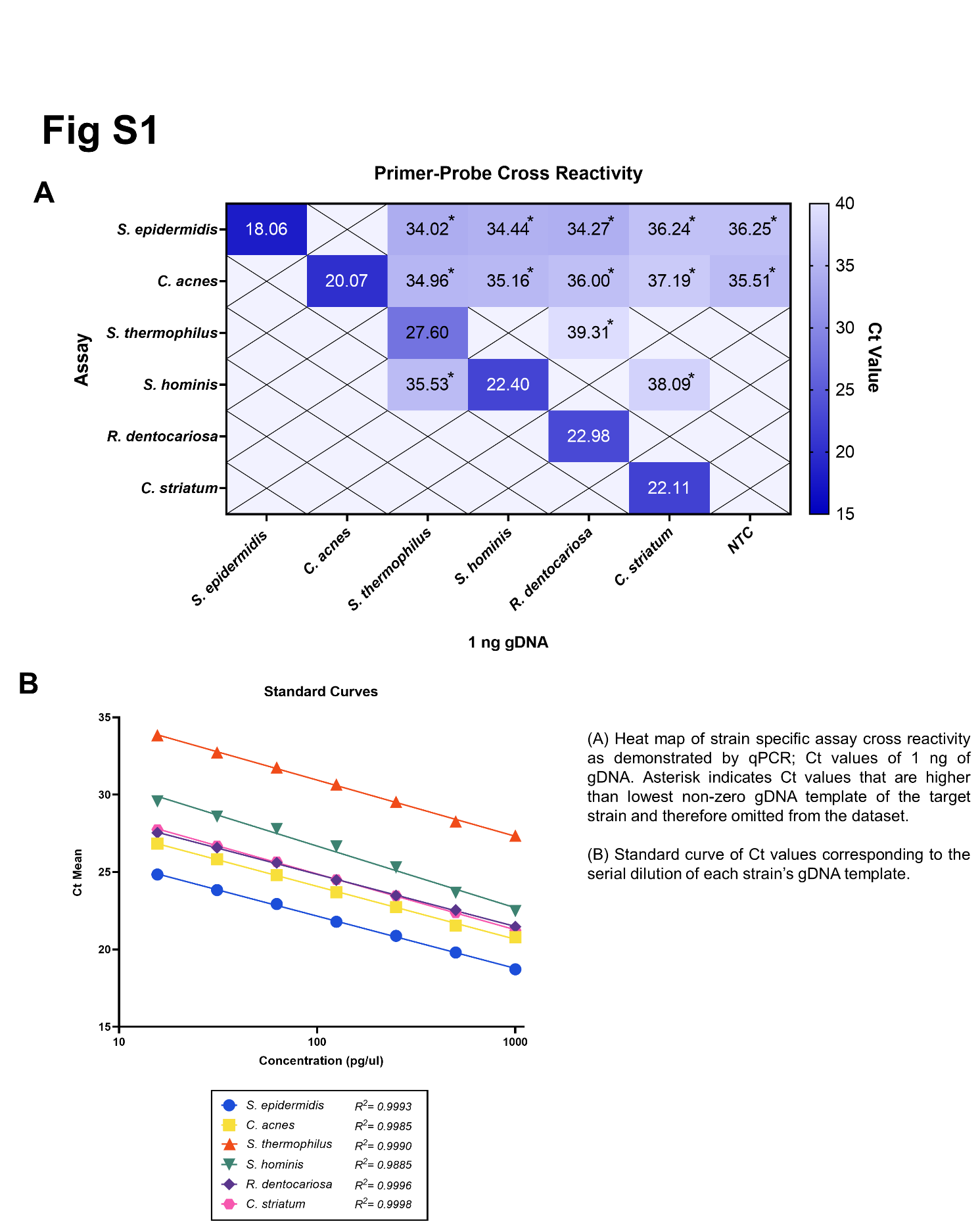


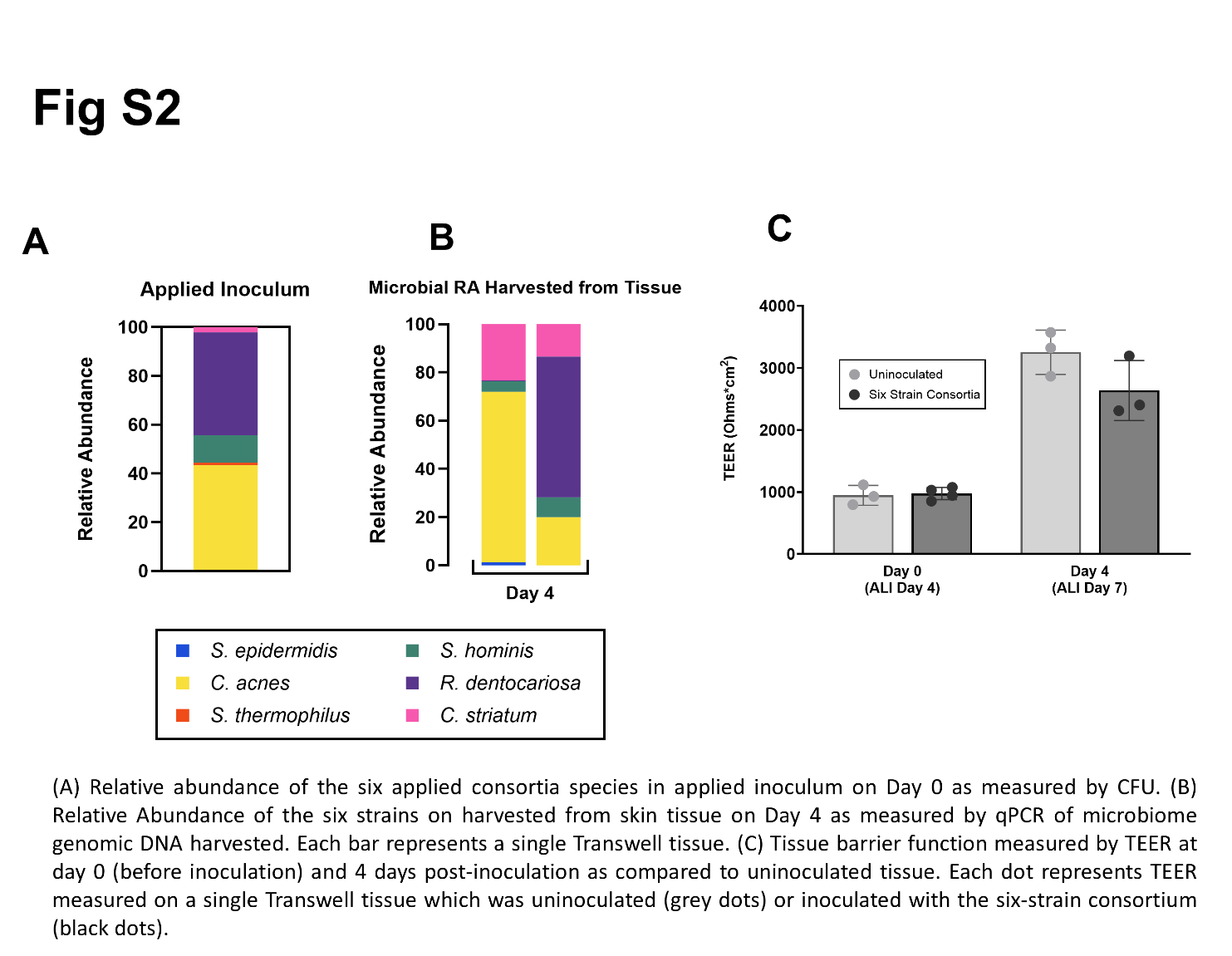


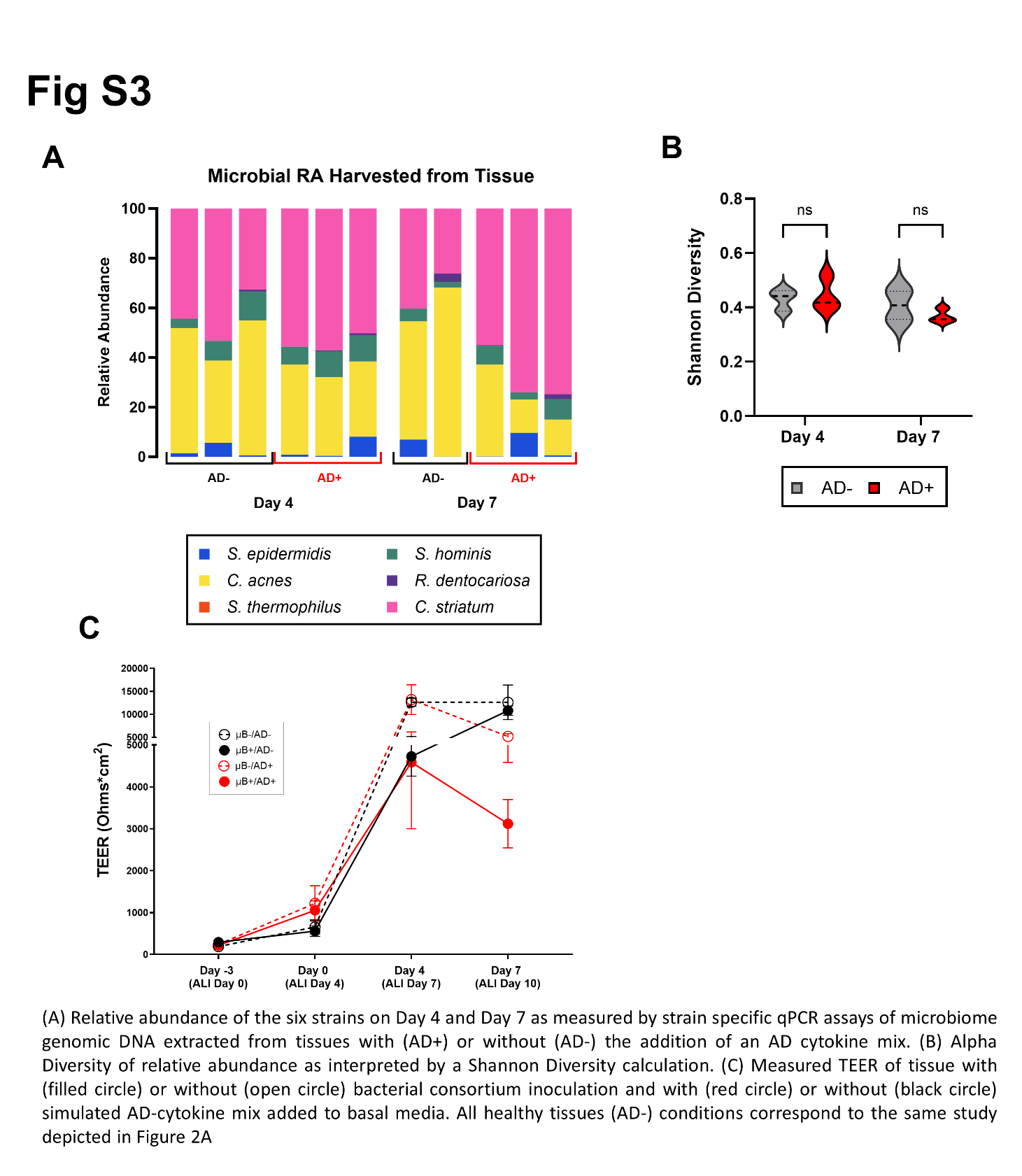


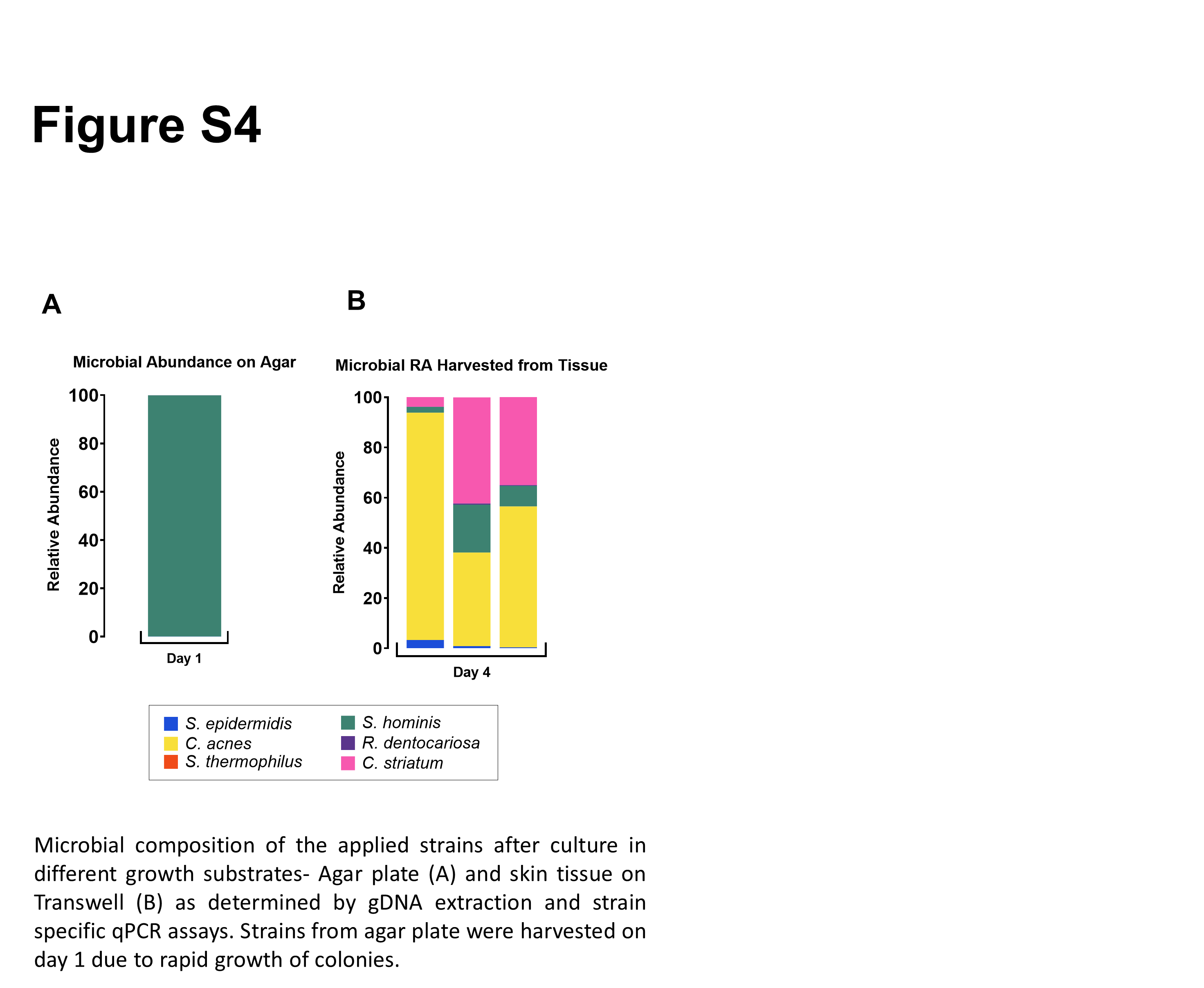

Supplement: Supplementary file 1 [file DataSheet1.docx]
